# Protective Effect of Adrenomedullin on Rat Leydig Cells from Lipopolysaccharide-Induced Inflammation and Apoptosis via the PI3K/Akt Signaling Pathway ADM on Rat Leydig Cells from Inflammation and Apoptosis

**DOI:** 10.1155/2016/7201549

**Published:** 2016-04-26

**Authors:** Pang-Hu Zhou, Wei Hu, Xiao-Bin Zhang, Wei Wang, Li-Jun Zhang

**Affiliations:** ^1^Department of Orthopedics, Renmin Hospital of Wuhan University, No. 238 Liberation Road, Wuhan, Hubei 430060, China; ^2^Department of Urology, Renmin Hospital of Wuhan University, No. 238 Liberation Road, Wuhan, Hubei 430060, China

## Abstract

This study was carried out to investigate whether ADM can modulate LPS-induced inflammation and apoptosis in rat Leydig cells. Leydig cells were treated with ADM before LPS-induced cytotoxicity. We determined the concentrations of ROS, MDA, GSH, LDH, and testosterone and the MMP. The mRNA levels of IL-1, IL-6, iNOS, and COX-2 were obtained, and the concentrations of IL-1, IL-6, NO, and PGE2 were determined. Apoptosis was assessed by TUNEL and detection of DNA fragmentation. The levels of mRNA and protein were determined for Bcl-2, Bax, caspase-3, and PARP. The protein contents for total and p-Akt were measured. ADM pretreatment significantly elevated the MMP and testosterone concentration and reduced the levels of ROS, MDA, GSH, and LDH. ADM pretreatment significantly decreased the mRNA levels of IL-1, IL-6, iNOS, and COX-2 and the concentrations of IL-1, IL-6, NO, and PGE2. LPS-induced TUNEL-positive Leydig cells were significantly decreased by ADM pretreatment, a result further confirmed by decreased DNA fragmentation. ADM pretreatment decreased apoptosis by significantly promoting Bcl-2 and inhibiting Bax, caspase-3, and PARP expressions. The LPS activity that reduced p-Akt level was significantly inhibited by ADM pretreatment. ADM protected rat Leydig cells from LPS-induced inflammation and apoptosis, which might be associated with PI3K/Akt mitochondrial signaling pathway.

## 1. Introduction

Leydig cells are exclusively responsible for testosterone production in males; testosterone is essential for male fertility and the maintenance of spermatogenesis [1]. Inflammation is an adaptive response to tissue malfunction or homeostatic imbalance by the body to ensure the removal of noxious stimuli and promote the healing process of repaired damaged tissue [2]. Under certain conditions, inflammation can be triggered by various inducers, including microbial infection, tissue injury, or toxic compounds [3]. Bacterial lipopolysaccharide (LPS), an active component of Gram-negative bacterial cell walls, can induce acute inflammation which is implicated in infection-associated testicular tissue damage, including testicular steroidogenesis and disrupted spermatogenesis [4]. Inflammatory resolution is increasingly viewed as an active process involving a number of key mediators, with the dysregulation of this process possibly predisposing individuals to the development of chronic inflammatory diseases [5]. In the normal resolution of inflammatory reactions, apoptosis is acknowledged to play a crucial role, whereas dysregulation in the induction of apoptosis by enhanced reactive oxygen species (ROS) production could also result in excessive apoptosis identified in the pathogenesis of human inflammatory diseases [6]. Although the cellular mechanisms involved in maintaining a constant population of Leydig cells are not well understood, apoptosis is thought to play an important role in the regulation of these cells. However, increased apoptosis can also cause a decline in testosterone production that can impair fertility [7].

Adrenomedullin (ADM) is a potent, 52-amino-acid hypotensive peptide originally isolated from human pheochromocytoma [8]. Besides its major role in the control of vascular function, ADM can mediate multifunctional responses in cell culture and animal systems, particularly regulation of cell proliferation, differentiation, and apoptosis [9, 10]. ADM can reduce the inflammatory response by downregulating the production of inflammatory mediators, including cytokines, chemokines, and free radicals [11]. ADM2, a member of the ADM peptide family, plays an important protective role in steroidogenesis in hydrogen peroxide-treated rat Leydig cells under primary culture [12]. The gene expression of ADM and its receptor component proteins has been reported in rat testis, isolated rat Leydig cells, and Sertoli cells [13–15]. The level of immunoreactive ADM in the testis is significantly lower than that in the adrenal gland and the epididymis, although the ADM mRNA levels in these tissues are similar [16, 17]. This discrepancy between the peptide contents and mRNA levels suggests that ADM may be actively secreted by the testis, as reported in other tissues [16, 17]. Therefore, many works have explored the gene expression of endogenous ADM and its receptor component proteins in the Leydig and Sertoli cells of rat testes. However, the effect of exogenous ADM on the number and function of Leydig cells by inhibiting inflammation and apoptosis has not been investigated.

Phosphatidylinositol 3-kinase/protein kinase B (PI3K/Akt) signaling pathway is a major signaling cascade that promotes cell survival and proliferation [18]. Among its various functions, activated PI3K/Akt signaling interferes with mitochondrial outer membrane permeabilization, thereby suppressing cell death [19]. Activated Akt translocates from the cell membrane to the cytoplasm and nucleus, where it can phosphorylate, activate, or suppress many downstream targets to regulate various cellular functions [20]. An appropriate Bcl-2/Bax balance is key to normal mitochondrial function [21]. Phosphorylated PI3K/Akt inhibits proapoptotic Bax activity but promotes antiapoptotic Bcl-2 release [22]. Therefore, PI3K/Akt signaling pathway may play a crucial role in regulating mitochondrial pathway-induced cell apoptosis.

In this study, we investigated whether ADM could attenuate LPS-induced inflammation and apoptosis by establishing an in vitro model of rat Leydig cells. We also explored the underlying mechanism of the protective role of ADM on Leydig cells by studying changes in the PI3K/Akt signaling pathway.

## 2. Materials and Methods

### 2.1. Reagents

Rat ADM (1-50) was purchased from Phoenix (Belmont, CA, USA). LPS from* Escherichia coli*, serotype (O127:B8), and LY294002 were obtained from Sigma (St. Louis, MO, USA). Dulbecco's modified Eagle's medium with the Hams F-12 nutrient mixture (at a 1 : 1 ratio; DMEM-F12), collagenase type IV, Percoll, Trypan Blue, bovine serum albumin (BSA), fetal bovine serum (FBS), 6-diamidino-2-phenylindole dihydrochloride (DAPI), and penicillin/streptomycin were obtained from Gibco (Grand Island, NY, USA). Cell Counting Kit-8 (CCK-8) was purchased from Dojindo Laboratories (Kumamoto, Japan). An in situ cell apoptosis detection kit was purchased from Roche Diagnostics (East Sussex, UK). TRIzol regent was acquired from Genei. Caspase-3 colorimetric assay kits were purchased from R&D Systems (Minneapolis, MN, USA). High capacity cDNA reverse transcription kit with RNase inhibitor and Power SYBR Green PCR master mix were purchased from Applied Biosystems (Foster City, CA), whereas primers for real-time PCR were obtained from Integrated DNA Technologies. Rabbit polyclonal antibodies for Bcl-2, Bax, caspase-3, and polyadenosine diphosphate-ribose polymerase (PARP) and rabbit monoclonal antibodies for Akt, phospho-Akt, and *β*-actin were obtained from Cell Signaling Technology (Beverly, MA, USA). All other chemicals used in this study were of analytical grade and obtained from Sigma (St. Louis, MO, USA).

### 2.2. Animals

Adult Sprague-Dawley rats approximately 90 days old and weighing approximately 400 g were purchased from the Experimental Animal Center of Wuhan University, China. The rats were individually maintained under standard conditions of controlled temperature (22 ± 1°C), lighting (12 h light : 12 h darkness), and humidity (50 ± 10%) with food and water available ad libitum and were used before they reached 120 days. The experimental animal procedures followed the national guidelines and protocols of the National Institutes of Health and were approved by the Local Ethics Committee for the Care and Use of Laboratory Animals of Wuhan University.

### 2.3. Leydig Cell Isolation and Purification

Isolation and purification of rat Leydig cell-enriched preparations were performed as previously described [23, 24], with modifications. Briefly, eight rats were euthanized with isoflurane, followed by cervical dislocation for each isolation event. Testes were dissociated from the scrotum and decapsulated without breaking the seminiferous tubules under aseptic conditions. Subsequently, testes were cut into small pieces and then placed in 50 mL plastic tubes (two testes per tube). Next, these were digested in 10 mL DMEM-F12 containing 0.25 mg/mL collagenase in a thermostatic shaking water bath with constant agitation (80 cycles/min) at 37°C for 45 min until the seminiferous tubules were separated. After incubation, the enzyme was diluted with collagenase-free DMEM-F12 until a total volume of 50 mL was reached. The tubes were allowed to settle for 10 min at room temperature without mixing. Tubules were washed again to detach the interstitium, and the two cell supernatants were combined. The resulting supernatant containing Leydig cells was filtered through a double layer of 100 *μ*m nylon mesh (Spectrum, Rancho Dominguez, California) and transferred to sterile centrifuge tubes. The cells were collected by centrifugation at 2500 ×g for 10 min at 4°C. After discarding the supernatant, the pellet obtained was resuspended in 2 mL of DMEM-F12 representing a crude testicular interstitial cell suspension. Discontinuous Percoll gradients were used to obtain purified Leydig cells from this crude preparation. The Leydig cell suspension was loaded on top of a discontinuous Percoll gradient (5%, 30%, 58%, and 70% Percoll in DMEM/F-12 medium) and then centrifuged at 800 ×g for 30 min at 4°C. After centrifugation, most of the purified Leydig cells were observed in the third Percoll gradient. These Leydig cells were carefully collected using a Pasteur pipette, transferred to centrifuge tubes containing DMEM-F12, and centrifuged at 800 ×g for 20 min at 4°C. The resulting supernatant was discarded. Percoll was removed by dilution with a Percoll buffer and centrifugation.

Leydig cell viability was estimated by measuring the percentage of cells that excluded Trypan Blue staining in accordance with the manufacturer's protocol. Briefly, isolated Leydig cells and an equal volume of 0.4% Trypan Blue were combined and incubated for 10 min at room temperature. After incubation, an aliquot of cells was loaded into a hemacytometer chamber for cell counting, and the numbers of nonviable (stained) and viable (excluded) cells were counted. Viability was calculated as the percentage of viable cells divided by the total cell count. Leydig cells with at least 95% viability were used for the subsequent experiments.

Leydig cell enrichment was assessed as previously described [25], with modifications. Briefly, the Leydig cell suspension was smeared on slides, dried at room temperature, incubated at 22°C for 90 min, and then washed with deionized water. The positive cells were stained with dehydroepiandrosterone (0.1% BSA, 1.5 mmol/L NAD^+^, 0.25 mmol/L nitroblue tetrazolium, and 0.2 mmol/L dehydroepiandrosterone in Ca^2+^- and Mg^2+^-free phosphate buffer solution (PBS), pH 7.4). The percentage of positively stained cells was determined under the microscope. Leydig cells showed intense staining and were 90% more enriched. Depending on the isolation, the yield per isolation from 16 testes ranged from 24 × 10^6^ to 32 × 10^6^ Leydig cells.

### 2.4. Cell Culture and Experimental Design

Leydig cells were cultured in 6-well plates (1 × 10^6^ cells/well) for a total volume of 2 mL DMEM-F12 containing 3% FBS, 24-well plates (1.25 × 10^5^ cells/well) for a total volume of 1 mL DMEM-F12 containing 3% FBS, or 96-well plates (1 × 10^4^ cells/well) for a total volume of 200 *μ*L DMEM-F12 containing 3% FBS. The cells were incubated at 37°C for 24 h under 5% CO_2_ and 95% air. At the end of incubation, the FBS medium was removed, and the cells were incubated with serum-free medium for 1 h before the onset of experimental treatments.

To determine the time- and dose-dependent effects of ADM, cells were cultured in 96-well plates with 200 *μ*L serum-free medium in the presence of 100 nM ADM for various times (0, 6, 12, 18, and 24 h) or in the presence of various doses of ADM (0, 10, 50, 100, and 300 nM) for 12 h, as indicated. Cell viability was measured by a CCK-8 assay.

To explore the protective effect of ADM on LPS-induced cytotoxicity, cells were incubated in 96-well plates with 200 *μ*L serum-free DMEM with ADM for 2 h before adding 1 *μ*g/mL LPS. Cells were cultured in serum-free medium in the control group. The CCK-8 assay was performed to detect cell viability at 12 h after incubation.

For other experiments, the cells were cultured in 6-well plates with 2 mL serum-free medium or 24-well plates with 1 mL serum-free medium. The cells were divided into five groups: control (cells were cultured in serum-free medium alone), LPS (cells were in serum-free medium containing 1 *μ*g/mL LPS for 12 h), ADM alone (cells were cultured in serum-free medium containing 100 nM ADM without LPS for 12 h), ADM + LPS (cells were cultured in serum-free medium containing 100 nM ADM for 2 h followed by 12 h with 1 *μ*g/mL LPS), and inhibition (cells were cultured in serum-free medium containing 100 nM ADM and 10 *μ*mol/L LY294002 for 2 h followed by 12 h with 1 *μ*g/mL LPS).

### 2.5. CCK-8 Assay

Cell proliferation was detected using a CCK-8 assay kit in accordance with the manufacturer's instructions. After implementing the above-described experimental design, the culture medium was collected, and the Leydig cells were washed two times with 0.1 M PBS. Then, 10 *μ*L of CCK-8 reagent was added to each well and incubated at 37°C for 2 h. The WST-8[2-(2-methoxy-4-phenyl)-3-(4-phenyl)-5-(2,4-sulphobenzene)-2H-tetrazolium monosodium salt] in the reagent could be reduced to orange-yellow formazan by dehydrogenase, which was proportional to the number of viable cells. Absorbance at 450 nm was recorded using a microplate reader (Perkin Elmer, Waltham, MA, USA). A standard curve was designed using Leydig cell suspension with different dilution rates to calculate the viable cell numbers in each sample.

### 2.6. Testosterone Measurement by Enzyme Immunoassay (EIA)

The amount of testosterone in the culture supernatant was measured using a testosterone EIA kit in accordance with the manufacturer's protocol. The absorbance at 450 nm was read in a Wallac 1420 microplate reader (Perkin Elmer, Waltham, MA, USA). The testosterone in the culture supernatant was calculated using a concurrent standard curve. Testosterone concentrations were expressed as ng/mL and normalized against the control group.

### 2.7. Measurement of Intracellular Reactive Oxidative Species (ROS)

Intracellular ROS levels were measured through the molecular probe of 2′,7′-dichlorofluorescein diacetate (DCFH-DA) [26]. DCFH-DA, a fluorescent dye, is decomposed by cellular esterases to nonfluorescent dye DCFH, which is then oxidized to DCF. The amount of intracellular ROS was proportional to the intensity of DCF fluorescence. After the Leydig cells were treated in accordance with the experimental design, the culture medium was removed and the cells were further incubated with DCFH-DA (100 *μ*M) in the dark at 34°C for 30 min. The cells were then washed with PBS and resuspended in 200 *μ*L PBS, and then fluorescence was measured in a Wallac 1420 microplate reader (Perkin Elmer, Waltham, MA, USA) with an excitation wavelength of 488 nm and emission wavelength of 520 nm. The amount of intracellular ROS was proportional to the intensity of DCF fluorescence, and the fluorescence intensity was recorded directly to indicate the relative amount of ROS. The ROS levels were normalized against the control group.

### 2.8. Malondialdehyde (MDA) Measurement

MDA is a byproduct of the oxidative degradation of cell membrane lipids and serves as an index of the intensity of oxidative stress. It is referred to as thiobarbituric acid reactive substance (TBARS), which can be easily measured via a previously reported protocol [27]. The amount of MDA in the cell lysates was determined by the absorbance of TBARS at 532 nm in a Wallac 1420 microplate spectrophotometer (Perkin Elmer, Waltham, MA, USA). The cellular MDA content was calculated using a concurrent standard curve. The results were expressed as pmol/mL and normalized against the control group.

### 2.9. Determination of Lactate Dehydrogenase (LDH) Release

Cell membrane integrity was determined by analyzing the supernatant medium for LDH release using a TOX-7 LDH assay kit, as previously described [28]. Absorbance was measured with a Wallac 1420 microplate reader (Perkin Elmer, Waltham, MA, USA) at a wavelength of 490 nm. Background optical absorbance was measured at 690 nm and was subtracted from primary measurements for each well. LDH content in the medium was calculated using a concurrent standard curve. LDH concentrations were expressed as U/L and normalized against the control group.

### 2.10. Estimation of Reduced Glutathione (GSH)

GSH is an important cellular nonenzymatic antioxidant. The reduced GSH in cell lysates was determined as previously described [29]. Fluorescence was measured in a Wallac 1420 microplate reader (Perkin Elmer, Waltham, MA, USA) where the excitation and emission wavelengths were 350 and 420 nm, respectively. Cellular GSH content was calculated using a concurrent standard curve. The results were expressed as *μ*g/mg protein and normalized against the control group.

### 2.11. Measurement of Mitochondrial Membrane Potential (MMP)

MMP was determined using a cationic fluorescent dye tetramethylrhodamine methyl ester (TMRM), as previously described [30]. The fluorescence intensity of this dye is proportional to the magnitude of MMP. Leydig cells were incubated with 1 *μ*M TMRM for 20 min at 34°C and carefully washed with PBS containing BSA (0.1%) to remove the unincorporated dye. Fluorescence intensity was determined in a Wallac 1420 microplate spectrofluorimeter (Perkin Elmer, Waltham, MA, USA) with excitation wavelength of 550 nm and emission wavelength of 600 nm. MMP was expressed as TMRM fluorescence intensity and was normalized against the control group.

### 2.12. Determination of IL-1 and IL-6 Cytokine Concentrations

The concentrations of IL-1 and IL-6 in the culture supernatant were determined with commercial enzyme-linked immunosorbent assay (ELISA) kits in accordance with the manufacturer's instructions. The IL-1 and IL-6 levels in the culture supernatant were calculated using a concurrent standard curve. The cytokine concentrations were expressed as pg/mL and normalized against the control group.

### 2.13. Quantification of Nitric Oxide (NO)

Nitrite levels in culture medium were assessed by a Griess reaction, as previously described [31]. To measure nitrite levels in the medium, sample aliquots were mixed with an equal volume of Griess reagent and the absorbance was spectrophotometrically determined at the wavelength of 550 nm in a Wallac 1420 microplate spectrofluorimeter (Perkin Elmer, Waltham, MA, USA). Nitrite concentrations were determined relative to a standard curve derived from known concentrations (1−100 nmol/mL) of sodium nitrite. The results were expressed as nmol/mL and normalized against the control group.

### 2.14. Assay of Prostaglandin E2 (PGE2) Concentrations

PGE2 levels in the culture medium were investigated using a commercially available ELISA kit in accordance with the manufacturer's instructions. PGE2 concentrations were determined relative to a standard curve. The results were expressed as ng/mL and normalized against the control group.

### 2.15. Terminal Deoxynucleotidyl Transferase dUTP Nick-End Labeling (TUNEL) Staining

Cell apoptosis was assessed with a TUNEL assay kit in accordance with supplied protocol. Cells were observed under an inverted fluorescence microscope, and apoptosis signals were counted manually. Randomly selected fields were photographed, and representative pictures were chosen to count apoptotic cells and total cells under 200x magnification. Leydig cells were stained with DAPI at 37°C for 30 min. Apoptotic Leydig cells were recognized with dual TUNEL and DAPI staining. The rate of TUNEL-positive cells in each field was calculated.

### 2.16. Detection of DNA Fragmentation

Intracellular DNA fragmentation in the cell lysates was detected using a cell death detection ELISA kit in accordance with the manufacturer's protocol. In brief, the treated cell lysates and the mixture of peroxidase-conjugated anti-DNA and biotin-labeled antihistone were transferred onto a streptavidin-coated plate and incubated for 2 h at room temperature. The plate was then washed thoroughly and incubated with 2,2′-azino-di-(3-ethylbenzothiazoline-6-sulfonic acid)-diammonium salt for 5–20 min. The optical density was spectrophotometrically determined at 405 nm with a Wallac 1420 microplate reader (Perkin Elmer, Waltham, MA, USA).

### 2.17. Quantitative Real-Time Polymerase Chain Reaction (PCR)

Real-time PCR was conducted to quantify changes in the mRNA levels of different genes involved in the process of inflammation and apoptosis following the experimental design. The treated cells were dissolved in TRIzol reagent and total RNA was extracted in accordance with the manufacturer's instructions. RNA concentration was determined using a spectrophotometer at 260 nm, and purity was assessed by measuring the ratio of A260/A280. Purified RNA with an A260/A280 ratio between 1.8 and 2.0 was used in this study. All primers for quantitative real-time PCR are purchased from Sigma, and their information is shown in Table 1. Real-time PCR was performed in a 7900HT Fast Real-Time PCR system (Applied Biosystems, USA) by using SYBR Green chemistry. Each reaction was run in triplicate and performed under standard conditions (25 *μ*L reaction mixture consisting of 0.5 *μ*L 10 mM dNTP, 2.5 *μ*L 10x buffer (containing Mg^2+^), 1 *μ*L upstream primer (50 *μ*g/mL), 1 *μ*L downstream primer (50 *μ*g/mL), 4 *μ*L cDNA, and 1 U Taq enzyme) in 40 cycles consisting of the following steps: initial denaturation at 95°C for 5 min, followed by a set cycle of denaturation at 94°C for 10 s, and different annealing temperatures for each pair of primers (ranging between 53 and 62°C) for 10 s, extension at 72°C for 28 s, and final elongation at 72°C for 5 min. The generation of specific PCR products was subjected to the analysis of melting curve; all the gene expressions were normalized for expression of the housekeeping gene, *β*-actin, and expressed as the fold ratio compared with the control.

### 2.18. Protein Extraction and Western Blot

Total protein was extracted from Leydig cell monolayers as follows: treated samples were collected and washed twice with precooled PBS. After the PBS was removed, protein extraction reagents containing protease inhibitors were added. The lysate was collected and centrifuged at 12,000 ×g for 20 min at 4°C. The protein-containing supernatant was then stored at −20°C.

The protein concentration of whole cell was determined using the bicinchoninic acid (BCA) assay kit using BSA as the standard. After adjusting for equal amounts of total protein, protein mixtures were separated by sodium dodecyl sulfate-polyacrylamide gel electrophoresis and transferred to polyvinylidene difluoride membranes. After transfer, nonspecific binding sites of the membranes were blocked for 1 h at room temperature in PBS, pH 7.4, containing 5% nonfat dry milk, and then incubated overnight at 4°C with primary antibodies (Bcl-2, Bax, caspase-3, PARP, Akt, and phospho-Akt). To control for protein loading, the membranes were probed with an anti-*β*-actin antibody. Next, the membranes were incubated for 2 h at room temperature with horseradish peroxidase-conjugated secondary antibodies. The results were scanned using a gel imaging system (UVP Company, Upland, CA, USA), and densitometry measurements were performed with Image Lab software (Bio-Rad Laboratories, Hercules, CA, USA). Relative protein expression was normalized to *β*-actin and compared with control group.

### 2.19. Caspase-3 Activity Assay

Caspase-3 activity was measured using a commercial caspase-3 colorimetric assay kit in accordance with the manufacturer's instructions. Briefly, freshly isolated Leydig cells were harvested by centrifugation and incubated in lysis buffer on ice for 15 min after treatment in accordance with the experimental design. The lysate was then centrifuged at 12,000 ×g and 4°C for 20 min, and the protein content was determined using the BCA assay kit. The lysates (10 *μ*L) were incubated with 10 *μ*L of 0.2 mM Ac-DEVD-pNA in 80 *μ*L of reaction buffer at 37°C for 2 h. The samples were measured at 405 nm using a microplate reader (Perkin Elmer, Waltham, MA, USA). The fold increase in caspase-3 activity was normalized to the control group.

### 2.20. Statistical Analysis

All data were expressed as mean ± stand error of the mean (SEM) of the average of the three wells in each of the five experiments. Data were analyzed using SPSS version 19.0 (SPSS Inc., Chicago, IL, USA). Significant differences among the mean values of multiple groups were evaluated with one-way ANOVA followed by Student-Newman-Keuls' method. A two-sided *P* value < 0.05 was considered statistically significant.

## 3. Results

**Figure 1:**
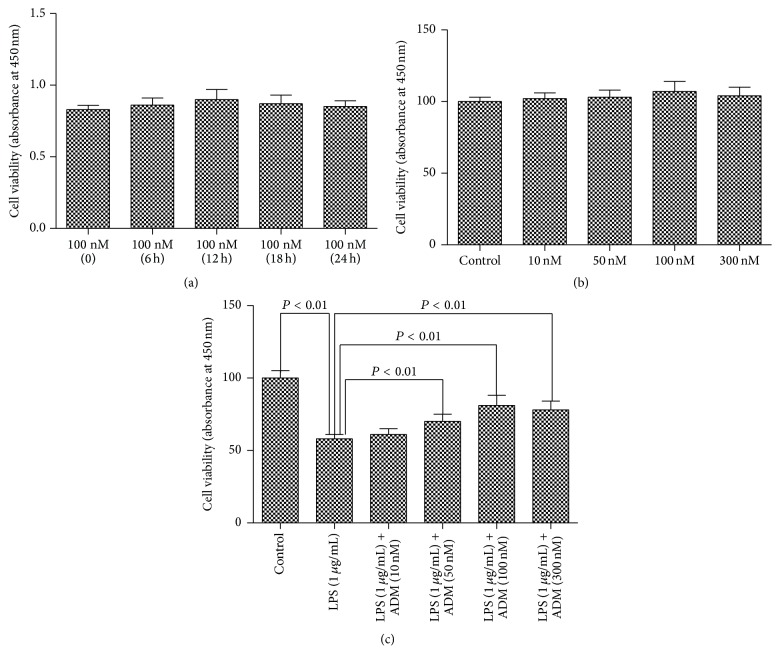
Effect of ADM on cell viability and LPS-induced damage of Leydig cells. Cell viability in different treatment groups was assessed using a CCK-8 assay. (a) Time course of effect of ADM (100 nM) on the viability of Leydig cells. (b) Dose-response effect of ADM (0, 10, 50, 100, or 300 nM) on the viability of Leydig cells after a 12-hour treatment. (c) ADM (0, 10, 50, 100, or 300 nM) was added for 2 h prior to a 12-hour treatment with LPS (1 *μ*g/mL). Data were obtained from five independent experiments performed in triplicate and expressed as mean ± SEM.

The effect of ADM on cell viability and the LPS-induced damage of Leydig cells are shown in Figure 1. No significant difference in absorbance was found among the five treated groups with different time courses (Figure 1(a)). However, the group at 12 h exerted a relatively stronger effect (Figure 1(a)). The absorbance did not significantly differ among the five treated groups with different concentrations of ADM, but the group with 100 nM ADM showed a relatively stronger effect (Figure 1(b)).

LPS significantly decreased cell viability (Figure 1(c)). However, LPS-induced cell damage was significantly reduced by the addition of ADM at concentrations of 50, 100, and 300 nM (*P* < 0.01). The protective effect did not significantly differ among the three groups, but the group with 100 nM ADM achieved a relatively stronger effect.

Thus, the following experiments were performed with a dose of 100 nM ADM at an exposure for 12 h unless otherwise stated.

**Figure 2:**
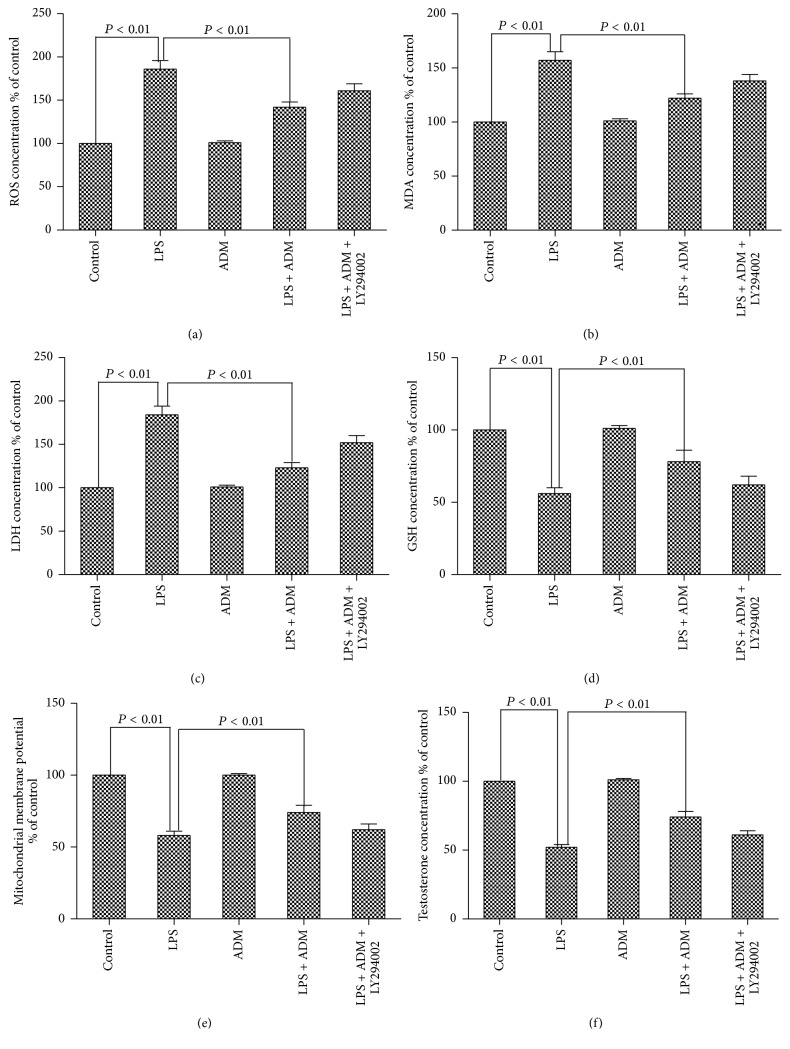
Effect of ADM on the concentrations of ROS, MDA, LDH, GSH, and testosterone and the MMP in the primary culture of Leydig cells. Leydig cells were treated with ADM (100 nM) or ADM (100 nM) + LY294002 (10 *μ*mol/L) for 2 h prior to a 12-hour treatment with LPS (1 *μ*g/mL). TMRM fluorescence intensity was analyzed on behalf of MMP. Data were obtained from five independent experiments performed in triplicate and expressed as mean ± SEM.

As shown in Figure 2, compared with the control group, LPS significantly increased the concentrations of ROS, MDA, and LDH and significantly decreased the levels of GSH and testosterone, as well as MMP (*P* < 0.01). When 100 nM ADM was added prior to LPS, the MMP and testosterone and GSH concentrations were significantly increased, whereas the ROS, MDA, and LDH levels were significantly decreased (*P* < 0.01). However, when the cells were pretreated with LY294002 (PI3K-specific inhibitor), the concentrations of ROS, MDA, and LDH were significantly higher, whereas MMP and GSH and testosterone levels were significantly lower (*P* < 0.01).

**Figure 3:**
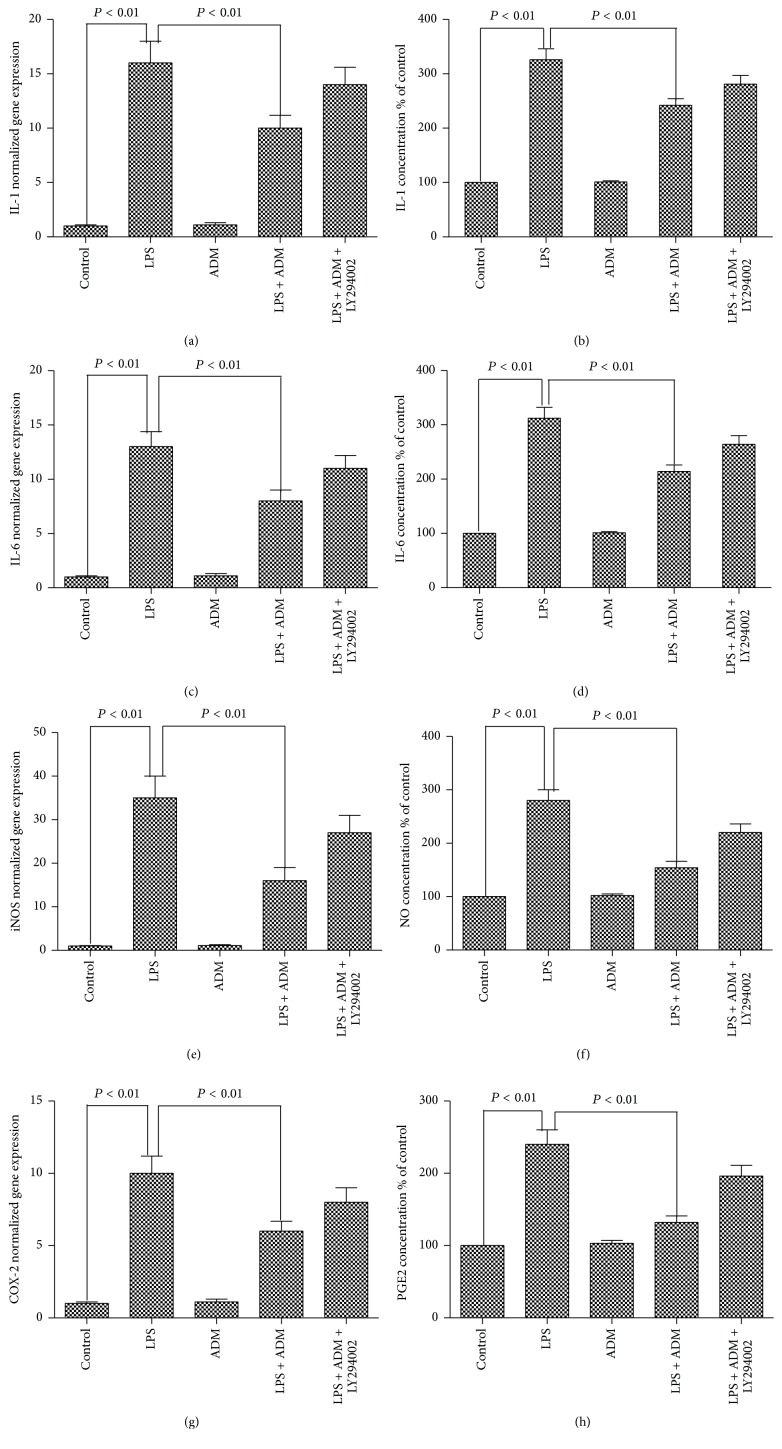
Effect of ADM on LPS-induced gene expressions of IL-1, IL-6, iNOS, and COX-2 and the production of IL-1, IL-6, NO, and PGE2. Leydig cells were treated with ADM (100 nM) or ADM (100 nM) + LY294002 (10 *μ*mol/L) for 2 h before a 12-hour treatment with LPS (1 *μ*g/mL). The normalized levels of gene expression are expressed as ratios of the copy number of the mRNA and that of *β*-actin cDNA. Culture media were analyzed for nitrite concentration on behalf of NO production. Data were obtained from five independent experiments performed in triplicate and expressed as mean ± SEM.

Figure 3 shows the effect of ADM on the LPS-induced gene expressions of IL-1, IL-6, iNOS, and COX-2 and the production of IL-1, IL-6, NO, and PGE2. Stimulation with LPS significantly increased the gene expressions of IL-1, IL-6, iNOS, and COX-2, as well as the production of IL-1, IL-6, NO, and PGE2 in the supernatant (*P* < 0.01). When 100 nM ADM was added prior to LPS, the gene expressions of IL-1, IL-6, iNOS, and COX-2 and the concentrations of IL-1, IL-6, NO, and PGE2 were significantly reduced (*P* < 0.01). However, the inhibitory effects of ADM on LPS-mediated gene expressions of IL-1, IL-6, iNOS, and COX-2 and concentrations of IL-1, IL-6, NO, and PGE2 were significantly blocked by LY294002 (*P* < 0.01).

**Figure 4:**
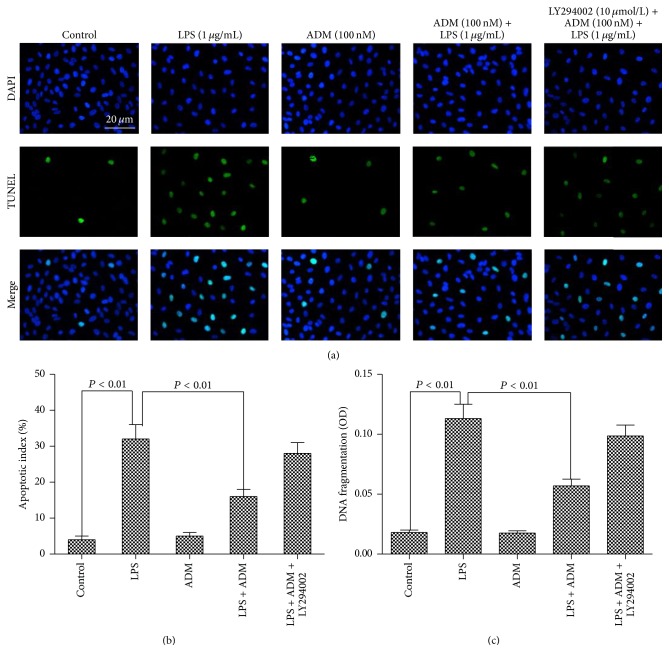
Antiapoptotic effect of ADM on LPS-induced apoptosis in Leydig cells. Leydig cells were treated with ADM (100 nM) with or without LY294002 for 2 h prior to a 12-hour treatment with LPS (1 *μ*g/mL). Cell apoptosis was assessed using TUNEL labeling and DAPI staining; DNA fragmentation was analyzed using ELISA detection (OD, optical density). Data were obtained from five independent experiments performed in triplicate and expressed as mean ± SEM.

The antagonistic effect of ADM on LPS-induced apoptosis is depicted in Figure 4. Stimulation with LPS significantly increased the percentage of TUNEL-positive cells compared with the control group (*P* < 0.01). When 100 nM ADM was added prior to LPS, the percentage of apoptotic cells was significantly reduced (*P* < 0.01). DNA fragmentation was also detected to analyze the apoptosis of the Leydig cells. The trend of the results was consistent with those of TUNEL staining. However, the antagonistic effect of ADM on LPS-mediated apoptosis was significantly blocked by LY294002 (*P* < 0.01).

**Figure 5:**
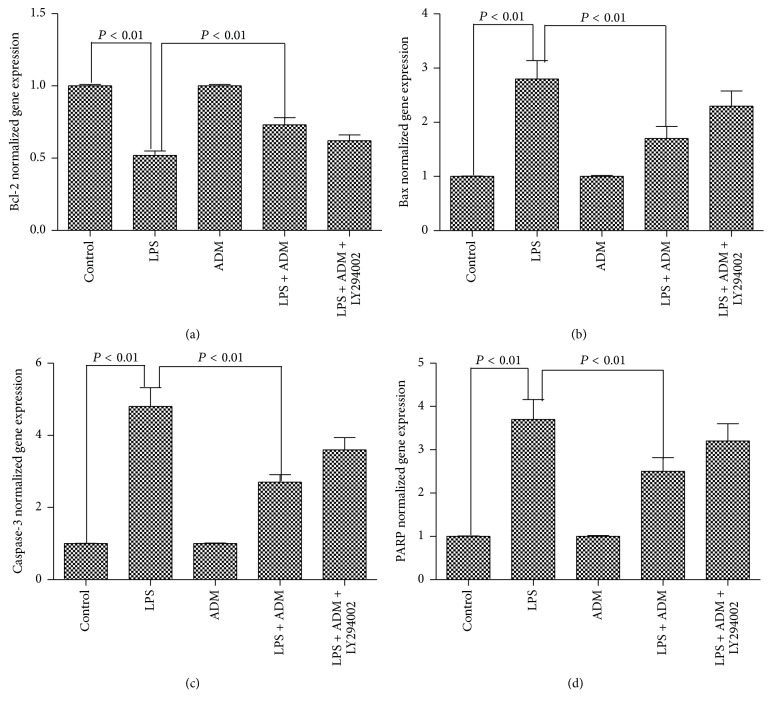
Effect of ADM on the LPS-induced gene expressions of Bcl-2, Bax, caspase-3, and PARP. Leydig cells were treated with ADM (100 nM) or ADM (100 nM) + LY294002 (10 *μ*mol/L) for 2 h before a 12-hour treatment with LPS (1 *μ*g/mL). The normalized levels of gene expression are expressed as ratios of the copy number of the mRNA and that of *β*-actin cDNA. Data were obtained from five independent experiments performed in triplicate and expressed as mean ± SEM.

Compared with the control group, the gene expression of Bcl-2 was significantly reduced by LPS, whereas the gene expressions of Bax, caspase-3, and PARP were significantly increased by LPS (*P* < 0.01) (Figure 5). When 100 nM ADM was added prior to LPS, the gene expression of Bcl-2 was significantly increased and the gene expressions of Bax, caspase-3, and PARP were significantly decreased (*P* < 0.01). However, the gene expression of Bcl-2 was significantly reduced, and the gene expressions of Bax, caspase-3, and PARP were significantly increased when the cells were pretreated with LY294002 (*P* < 0.01).

**Figure 6:**
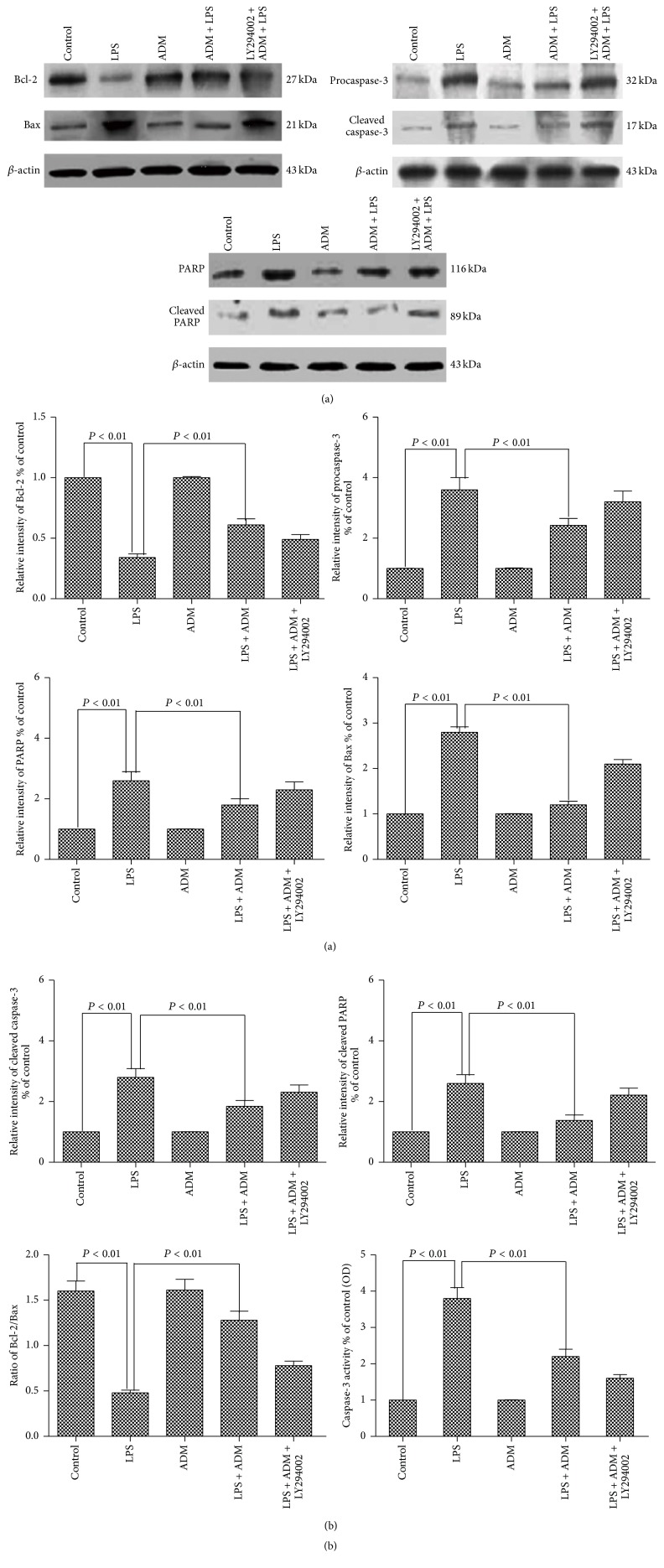
Effect of ADM on LPS-induced protein levels of Bcl-2, Bax, procaspase-3, cleaved caspase-3, PARP, and cleaved PARP and caspase-3 activity. Leydig cells were treated with ADM (100 nM) or ADM (100 nM) + LY294002 (10 *μ*mol/L) for 2 h prior to a 12-hour treatment with LPS (1 *μ*g/mL). Protein levels were detected by Western blot; caspase-3 activity was measured by spectrophotometry (OD, optical density). *β*-actin was used as internal reference. Data were obtained from five independent experiments performed in triplicate and expressed as mean ± SEM.

LPS caused a significant decrease in the protein level of Bcl-2 and significant increases in the protein levels of Bax, procaspase-3, cleaved caspase-3, PARP, and cleaved PARP compared with the control group (*P* < 0.01) (Figure 6). Moreover, the ratio of Bcl-2/Bax was significantly decreased, whereas caspase-3 activity was significantly increased (*P* < 0.01). When ADM was added prior to LPS, the protein level of Bcl-2 was significantly increased and the protein levels of Bax, procaspase-3, cleaved caspase-3, PARP, and cleaved PARP were significantly decreased (*P* < 0.01). The ratio of Bcl-2/Bax was also significantly increased, and the caspase-3 activity was significantly decreased (*P* < 0.01). However, the increased protein level of Bcl-2, the ratio of Bcl-2/Bax, and the decreased protein levels of Bax, procaspase-3, cleaved caspase-3, PARP, cleaved PARP, and caspase-3 activity by ADM pretreatment were significantly blocked by LY294002 (*P* < 0.01).

**Figure 7:**
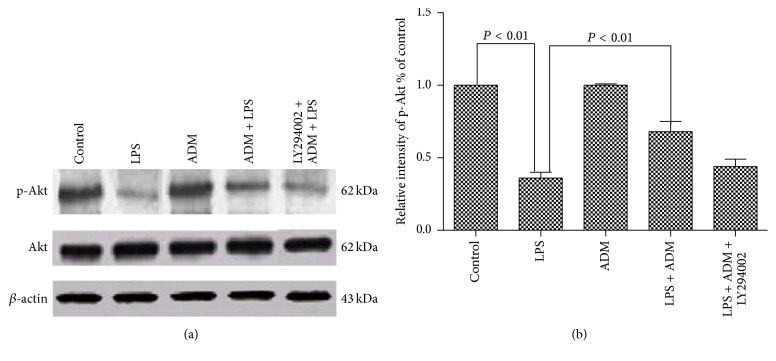
Effect of ADM on LPS-induced inhibition of Akt phosphorylation. Leydig cells were treated with ADM (100 nM) or ADM (100 nM) + LY294002 (10 *μ*mol/L) for 2 h before a 12-hour treatment with LPS (1 *μ*g/mL). *β*-actin was used as internal reference, and p-Akt levels were normalized to total Akt levels. Data were obtained from five independent experiments performed in triplicate and expressed as mean ± SEM.

Figure 7 shows the effect of ADM on LPS-induced inhibition of Akt phosphorylation. In protein extracts from Leydig cells, a reduced level of LPS-induced phospho-Akt was observed compared with the control group (*P* < 0.01) without altering total Akt level. The protein level of phospho-Akt was significantly increased by ADM pretreatment (*P* < 0.01). However, the increased protein level of phospho-Akt was significantly blocked by LY294002 (*P* < 0.01).

## 4. Discussion

The endogenous antioxidant potential to protect against ROS-induced podocyte injury may be possessed by ADM [32]. ADM induces the downregulation of inflammatory cytokines in cultured cells and downregulates inflammatory processes in a variety of different colitis models [33]. ADM plays an important role in regulating systemic inflammation and may be an important intrinsic factor for protecting against liver damage in LPS-induced endotoxemia [34]. Continuous infusion of ADM ameliorated the LPS-induced acute lung injury in rats and this beneficial effect of ADM on acute lung injury may be mediated by the inhibition of inflammation, hyperpermeability, and alveolar wall cell apoptosis [35].

In our study, the cell viability of Leydig cells upon ADM treatment was relatively the highest when ADM was at the concentration of 100 nM. Moreover, the protective effect of ADM on LPS-induced damage of Leydig cells was relatively the strongest when ADM was at the concentration of 100 nM. Data from the present study agree with those from previous reports [9, 36], although the latter works involved other cell types, such as osteoblasts and endothelial progenitor cells.

Overexpression of ADM2, which belongs to the ADM peptide family in the kidney, apparently reduces oxidative stress and suppresses inflammation and apoptosis [37]. In the current paper, ADM pretreatment improved steroidogenesis dysfunction by attenuating the LPS-induced excessive oxidative stress and ameliorating mitochondrial function. ADM possibly exerts a protective role in steroidogenesis and the mitochondrial function of Leydig cells by inhibiting LPS-induced excessive oxidative stress.

LPS can cause the activation of an acute inflammatory response in the testes accompanied by a significant decrease in testosterone production and disruption of spermatogenesis [38]. ADM2 can attenuate the increase in the gene expressions of IL-1*β* and IL-6, rescue spermatogenesis, and prevent the decrease in plasma testosterone levels caused by LPS [12]. Inflammatory agents such as NO and PGE2, which are, respectively, produced by iNOS and COX-2, may be involved in some part of the regulation of Leydig cell dysfunction [39, 40]. ADM significantly reduces the development of acute lung injury by downregulating a broad spectrum of inflammatory factors [41]. In the present study, ADM pretreatment significantly decreased the gene expressions of IL-1, IL-6, iNOS, and COX-2 and the production of IL-1, IL-6, NO, and PGE2. These findings suggest that LPS-induced uncontrolled inflammation in Leydig cells can be attenuated by ADM pretreatment. Therefore, ADM may act as an anti-inflammatory agent by inhibiting LPS-induced uncontrolled inflammation and further result in excessive oxidative stress.

Uncontrolled inflammation has been widely demonstrated to lead to apoptosis, which is acknowledged to play a crucial role in the pathogenesis of inflammatory diseases [6]. The antiapoptotic effect of ADM appears to be partly responsible for its beneficial effects on vascular endothelial cell apoptosis that occurs in late sepsis [42]. The current study showed that ADM pretreatment can significantly reduce the LPS-induced higher apoptotic percentage and DNA fragmentation of Leydig cells. These data suggest that ADM pretreatment may improve LPS-related apoptosis of Leydig cells by inhibiting uncontrolled inflammation from progressing to apoptosis.

The Bcl-2 gene family members, including Bax and Bcl-2, play an essential role in regulating apoptosis by forming hetero- and homodimers in the mitochondrial membrane, with the prevailing outcome depending on the ratio of Bcl-2 to Bax of apoptosis [21]. In our study, ADM pretreatment increased the gene expression and protein levels of Bcl-2 and decreased the gene expression and protein levels of Bax, which resulted in the increased ratio of Bcl-2/Bax. Our work demonstrated that ADM may inhibit the mitochondrial pathway to block LPS-induced Leydig cell apoptosis by reducing mitochondrial permeability. Caspase activation is the final process of the death signaling pathway, in which procaspase-3 is activated to caspase-3, the most important biomarker and executor of cell apoptosis. Caspase-3 then induces the hydrolysis of nucleic acids and cytoskeletal proteins [43]. PARP, a downstream target of caspase-3, can be cleaved in fragments of 89 and 24 kDa by an active form of caspase-3. This cleaved PARP loses the ability to participate in DNA repair, which results in apoptosis and is a useful hallmark of this type of cell death [44, 45]. Therefore, inhibiting extensive activation of caspase-3 and PARP may be the key to decreasing Leydig cell apoptosis. We found that ADM pretreatment significantly downregulated the expression levels of these genes in LPS-stimulated Leydig cells. The upregulated protein levels of procaspase-3, cleaved caspase-3, PARP, and cleaved PARP were also significantly inhibited by ADM pretreatments; likewise, increased caspase-3 activity was significantly reduced by ADM pretreatment. PARP activity is well known to increase with oxidative stress, which can lead to cellular dysfunction and necrosis [46]. Given these findings, we infer that the LPS-induced apoptosis of Leydig cells resulted from excessive oxidative stress due to uncontrolled inflammation. ADM could inhibit Leydig cell apoptosis that resulted from the excessive oxidative stress derived from LPS-induced uncontrolled inflammation by inhibiting caspase-3 activation. Thus, PARP could not be cleaved into fragments and retains the potential to recover the ability to participate in DNA repair. This effect may be a highly important antiapoptotic mechanism for ADM.

We further investigated whether the PI3K/Akt signaling pathway is involved in the anti-inflammatory and antiapoptotic activities of ADM in response to LPS in Leydig cells. We focused on the PI3K/Akt pathway because of its essential role in promoting survival in various cell types [47]. Our results demonstrated that ADM pretreatment decreased the activity of LPS, an effect that reduced Akt phosphorylation and was effectively blocked by a PI3K inhibitor (LY294002). These findings suggest a possible role for the PI3K/Akt signaling pathway in the ADM-mediated protection of Leydig cells exposed to LPS.

Several limitations to our study must be acknowledged. First, all of our experiments were performed in cell lines; in vivo studies and clinical trials have yet to be performed to verify our hypothesis. Second, all our data were acquired with ADM pretreatment experiments, which appears to be essential for permitting the protective role of the compound in our study. Third, a possible overestimation of the protective role of ADM cannot be excluded because of the effect of the endogenous ADM from Leydig cells.

## 5. Conclusions

In conclusion, our study showed that ADM inhibited LPS-induced inflammation and rat Leydig cell apoptosis under a mechanism possibly associated with the PI3K/Akt mitochondrial signaling pathway. Collectively, these results can be extrapolated to idiopathic male infertility in which an overwhelming proinflammatory microenvironment can result in excessive oxidative stress and further lead to apoptosis in the testis. ADM may protect the damaged testicular Leydig cell from apoptosis under pathological conditions such as oxidative stress resulting from uncontrolled inflammation.

## Figures and Tables

**Table 1:** Sequences of primers for the real-time PCR experiments.

Gene	Accession number	Sense	Sequence 5′ → 3′	Size (bp)
IL-1	NM_031512.2	F R	CATTGTGGCTGTGGAGAAG ATCATCCCACGAGTCACAGA	130

IL-6	NM_012589.1	F R	TCCTACCCCAACTTCCAATGCTC TTGGATGGTCTTGGTCCTTAGCC	79

iNOS	NM012611.3	F R	ACCAGTACGTTTGGCAATGG TCAGCATGAAGAGCGATTTCT	70

COX-2	NM017232.3	F R	CTTACAATGCTGACTATGGCTAC AAACTGATGCGTGAAGTGCTG	242

Bcl-2	NM001001280.1	F R	CCACCAAGAAAGCAGGAAACC GGCAGGATAGCAGCACAGG	129

Bax	NM017059.2	F R	TGGCAGCTGACATGTTTTCTGAC CGTCCCAACCACCCTGGTCT	195

Caspase-3	NM012922.2	F R	CGATTATGCAGCAGCCTCAA AGGAGATGCCACCTCTCCTT	118

PARP	NM013063.2	F R	TCTTTGATGTGGAAAGTATGAAGAA GGCATCTTCTGAAGGTCGAT	64

*β*-actin	NM031144.3	F R	CGTTGACATCCGTAAAGAC TGGAAGGTGGACAGTGAG	201

F: forward; R: reverse.
